# Case Report: Surgical management of traumatic giant coronary artery pseudoaneurysm with pericardial patch repair and ostium isolation

**DOI:** 10.3389/fcvm.2024.1462557

**Published:** 2024-11-08

**Authors:** Hongjia Ma, Hong Qian, Wei Meng

**Affiliations:** Department of Cardiovascular Surgery, West China Hospital, Sichuan University, Chengdu, China

**Keywords:** coronary artery aneurysm, coronary artery pseudoaneurysm, giant coronary artery pseudoaneurysm, surgical intervention, trauma

## Abstract

There is limited literature regarding cases of giant coronary artery aneurysms (GCAAs), and instances of giant coronary artery pseudoaneurysms caused by trauma are exceedingly rare. Here is a case presentation of an adult male who developed a giant coronary artery pseudoaneurysm following trauma. Successful surgical intervention was performed, involving repair of the aneurysmal opening with a pericardial patch and isolation of the right coronary artery ostium into the aortic root. One month postoperatively, a follow-up transthoracic echocardiogram revealed thrombotic occlusion within the residual lumen of the right coronary artery aneurysm, with contiguous echogenicity extending from the aortic sinus to the right coronary artery.

## Introduction

The notion of coronary artery aneurysm was originally postulated by Morgagni in 1761 (1). Presently, it is delineated as an expansion of the vascular structure with a diameter ≥1.5 times that of the adjacent normative vessel. Infrequently, this dilation may reach dimensions warranting classification as a giant coronary artery aneurysm, a concept subject to ongoing debate concerning precise criteria. Diverse definitions have been proposed by scholars, delineating aneurysms exceeding diameters of 20 mm, 40 mm, or 50 mm, or those surpassing fourfold the reference vessel diameter (2). Literature suggests that merely 0.02% of coronary artery aneurysms meet the stringent criteria for classification as giant coronary artery aneurysms (3, 4). The etiology of these aneurysms is multifaceted, with atherosclerosis emerging as the predominant cause, Coronary artery pseudoaneurysms are rare and often occur after catheter-based interventions, surgical complications, blunt trauma, or infection (5). Herein, we present a case involving an adult male patient diagnosed with a giant coronary artery pseudoaneurysm subsequent to trauma, who underwent successful surgical intervention.

## Case presentation

The patient is a 54-year-old male who experienced chest pain after colliding with a car while riding a motorcycle, resulting in a 16-h coma before presenting to our emergency department. There was no primary loss of consciousness after the injury, no evidence of open chest trauma, and no respiratory distress. He denies any family history of genetic diseases or past cardiac history, previous transthoracic echocardiography did not mention any abnormalities. Following initial treatment at a local hospital, his condition deteriorated gradually, leading to altered mental status, prompting endotracheal intubation and assisted ventilation with a mechanical ventilator.

Upon admission, vital signs were stable, and a computed tomography scan of the head revealed no significant abnormalities. Left ventricular ejection fraction was measured at 71%. The preoperative electrocardiogram indicated mild ST-T segment depression in the interventricular septum and lateral wall, with a peak troponin-T level of 872.0 ng/L.

**Figure 1:**
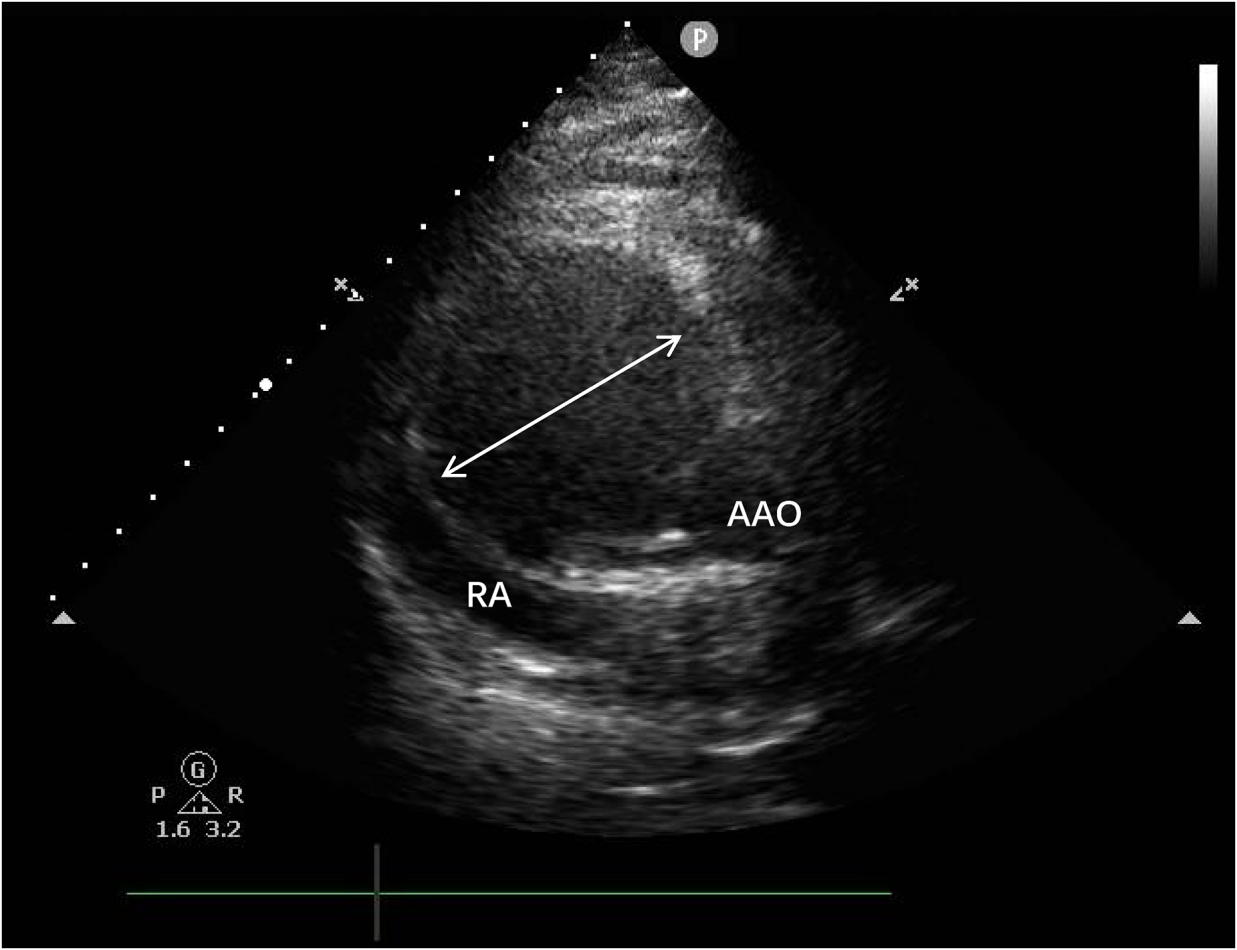
A tumor-like abnormal hypoechoic area (white arrow) measuring approximately 70 mm × 66 mm × 75 mm located about 1 cm from the aortic valve annulus, extending outward from the sinus. RA, right atrium; AAO, ascending aortat.

**Figure 2:**
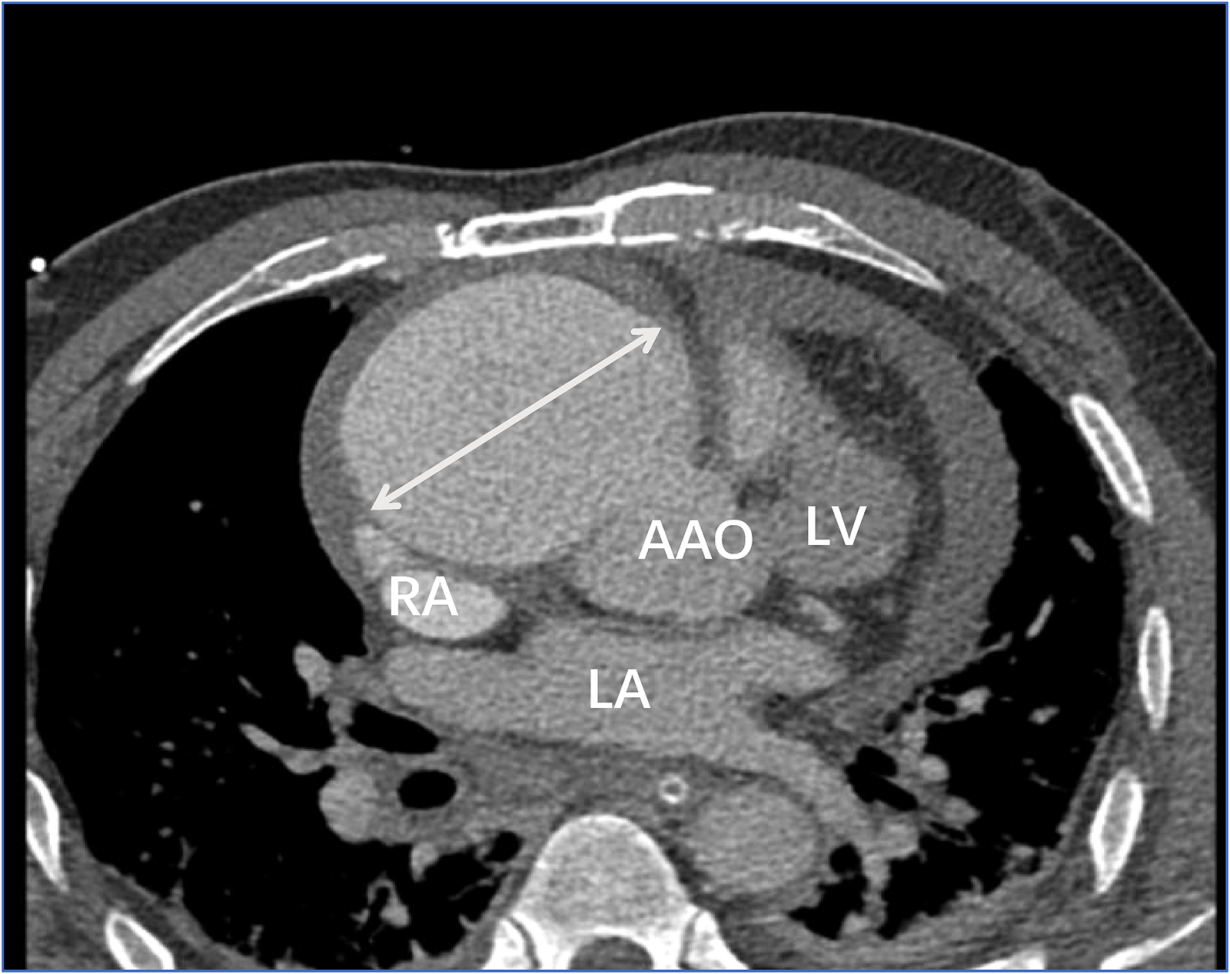
Contrast-enhanced computed tomography angiography confirmed a spherical dilatation (6.0 cm × 7.5 cm) in the right anterior aspect of the ascending aorta (white arrow). LA, left atrium; LV, left ventricle; RA, right atrium; AAO, ascending aorta.

Transthoracic echocardiography (Figure 1) revealed a tumor-like abnormal hypoechoic area measuring approximately 70 mm × 66 mm × 75 mm located about 1 cm from the aortic valve annulus, extending outward from the sinus. This mass communicated with the aorta through a 15 mm × 9 mm defect, with blood flow directed from the aorta to the right coronary artery. Contrast-enhanced computed tomography angiography (Figure 2) confirmed a spherical dilatation (6.0 cm × 7.5 cm) in the right anterior aspect of the ascending aorta. A defect measuring approximately 1.1 cm in diameter was seen in the anterior aspect of the ascending aorta, connecting with the spherical dilation. Compression was noted on the ascending aorta root, aortic sinuses, and right atrium.

**Figure 3:**
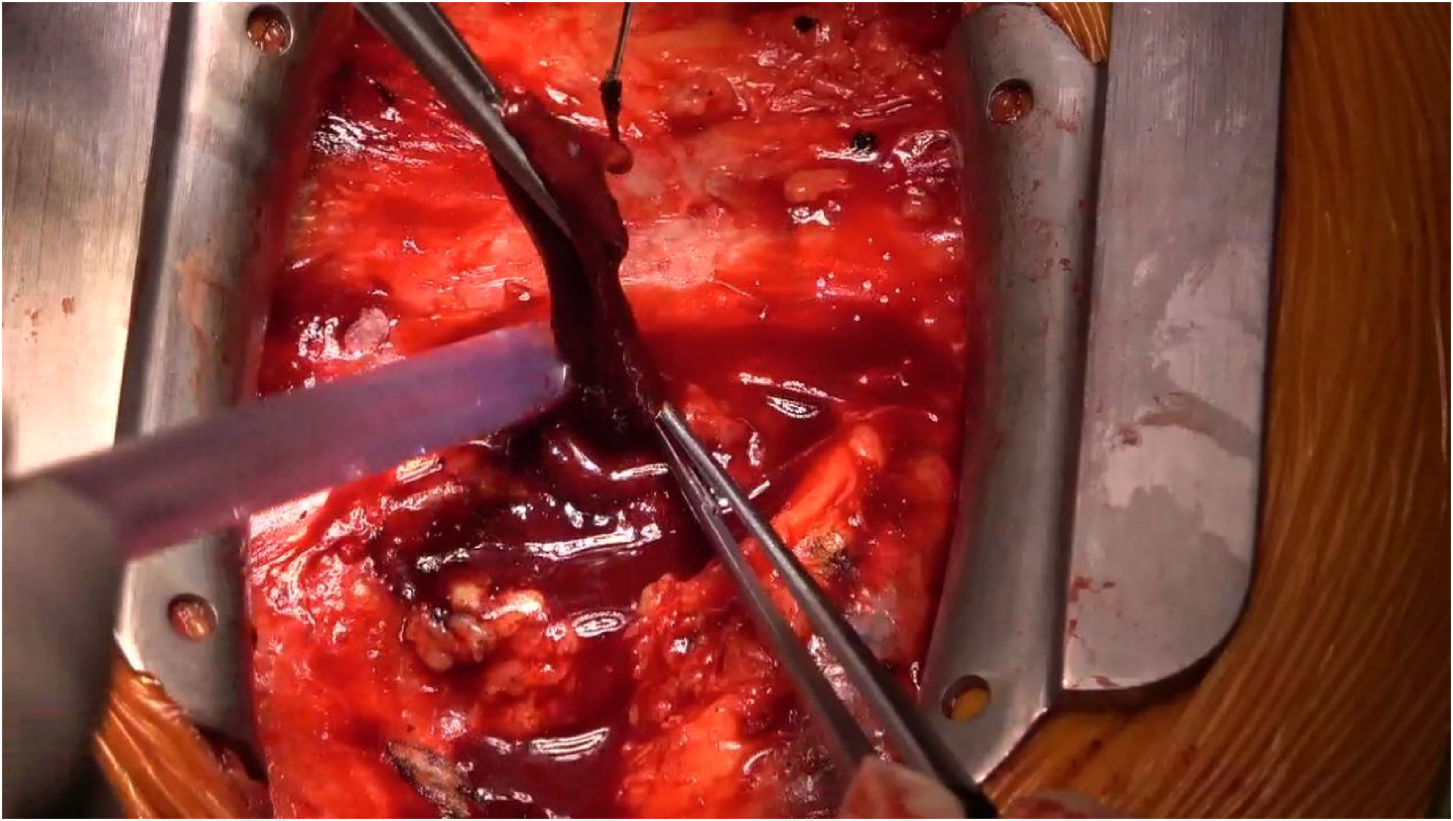
Within the pericardium, there is approximately 100 mL of dark red, old blood clots, along with partial thrombus formation.

**Figure 4:**
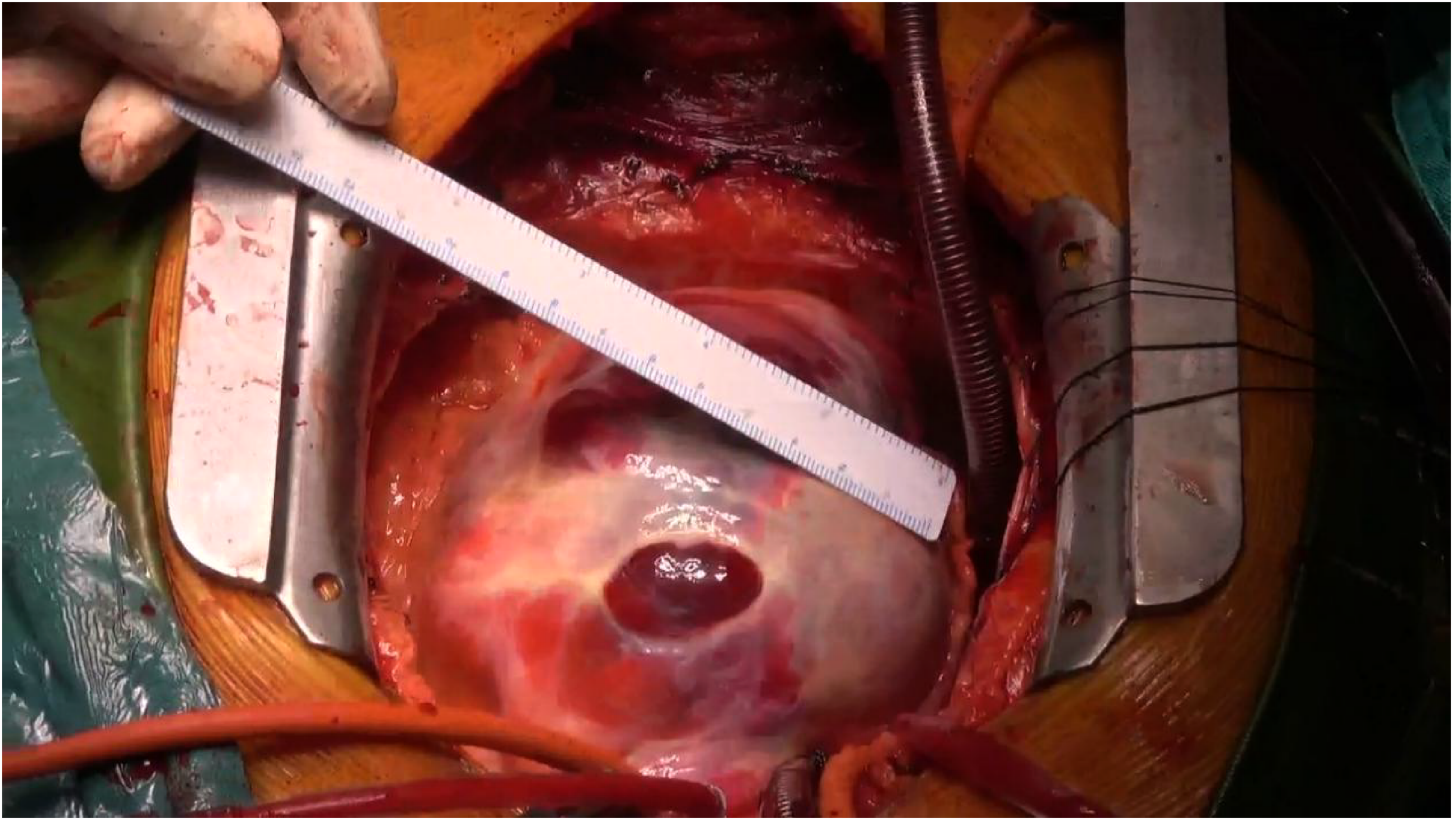
In the aortic root and atrioventricular groove, there is evident presence of a substantial mass, measuring approximately 12 cm × 10 cm.

**Figure 5:**
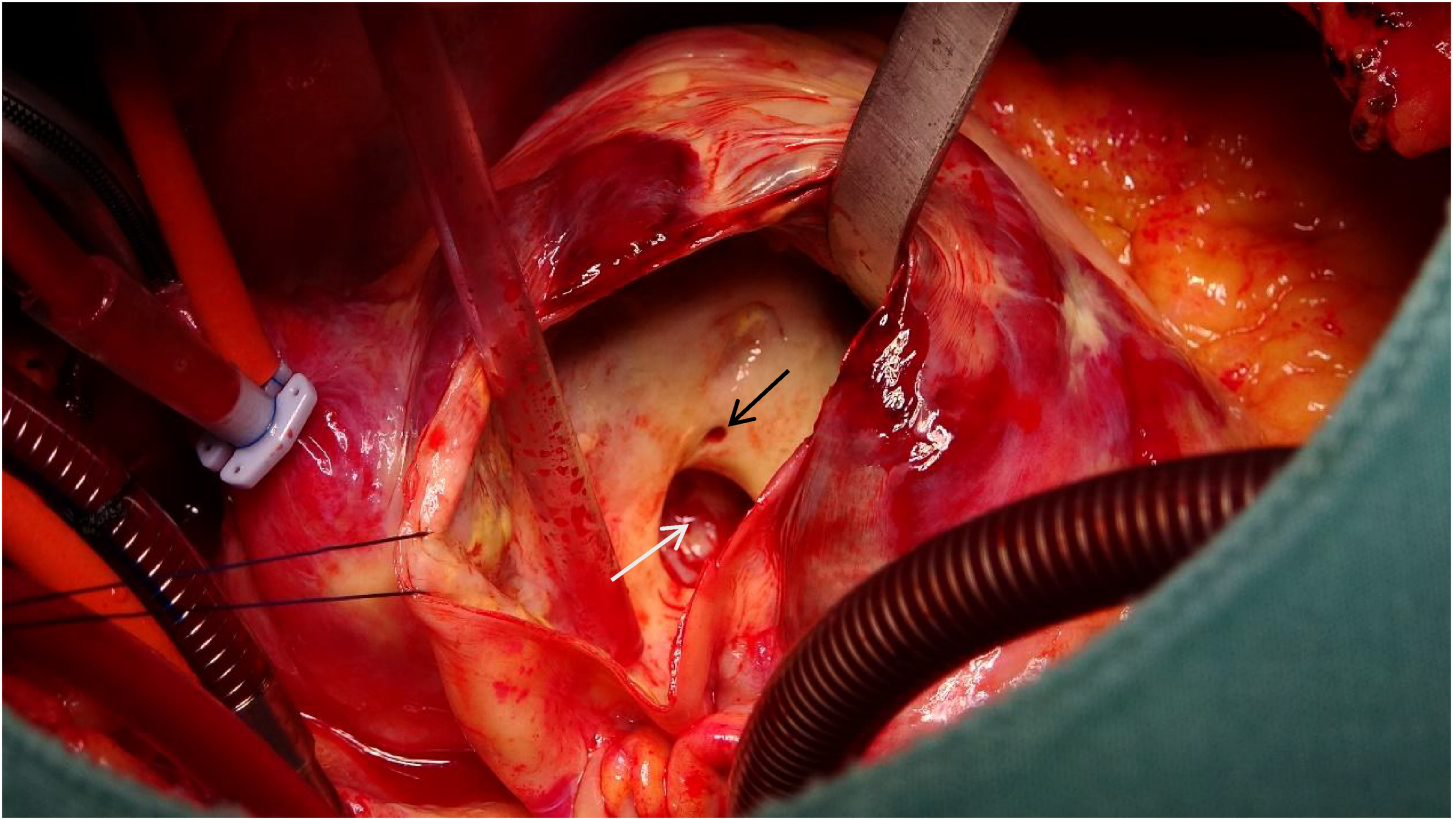
A 1.5 cm × 2 cm communication between the right coronary sinus wall and the aneurysm (white arrow), with the right coronary artery ostium (black arrow) positioned 5 mm proximal to the opening.

**Figure 6:**
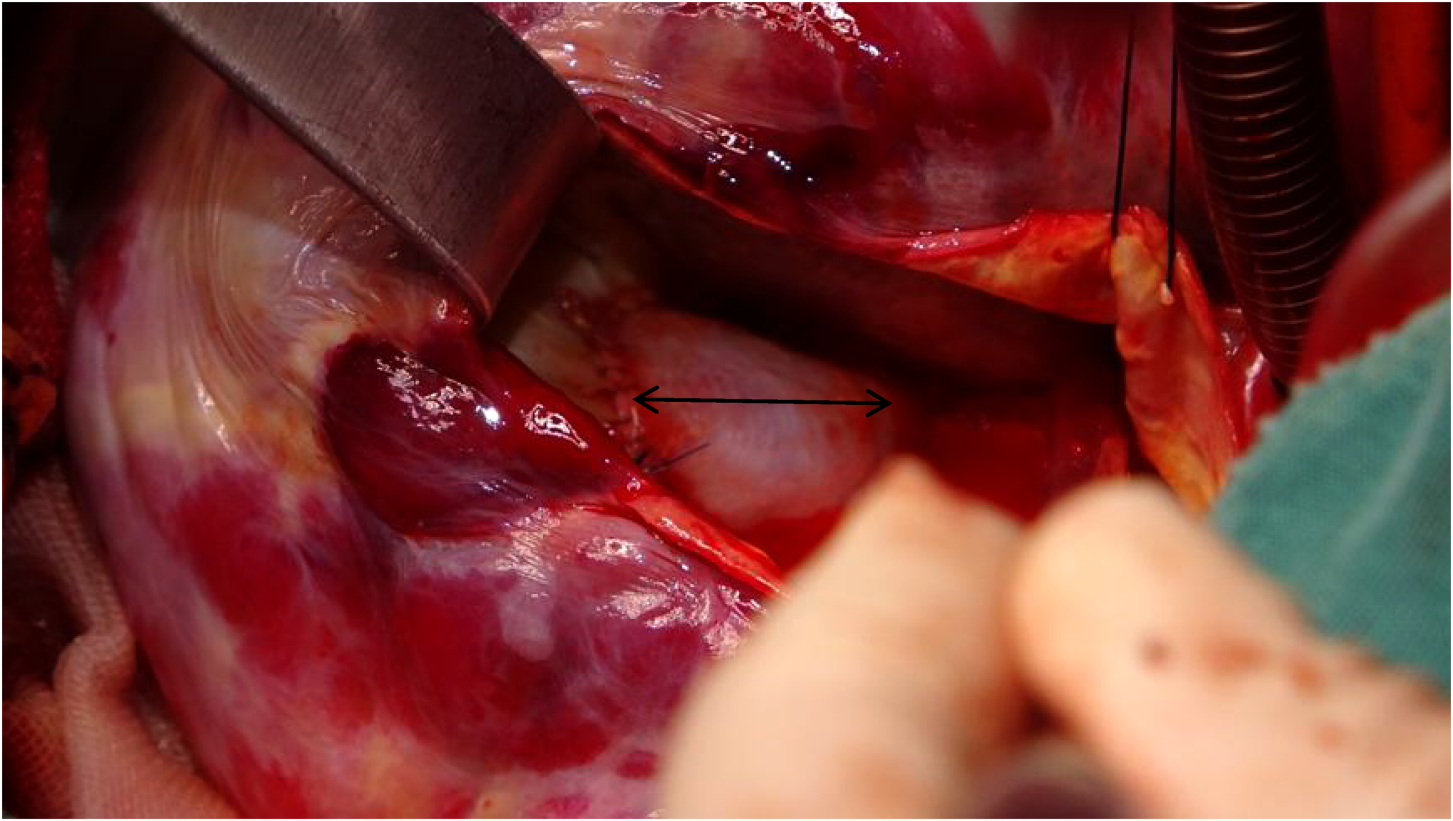
Utilizing a 2.5 cm × 2 cm pericardial patch (black arrow) to close the passage between the aortic root and the aneurysm, while resecting the ostium of the right coronary artery into the aortic root.

**Figure 7:**
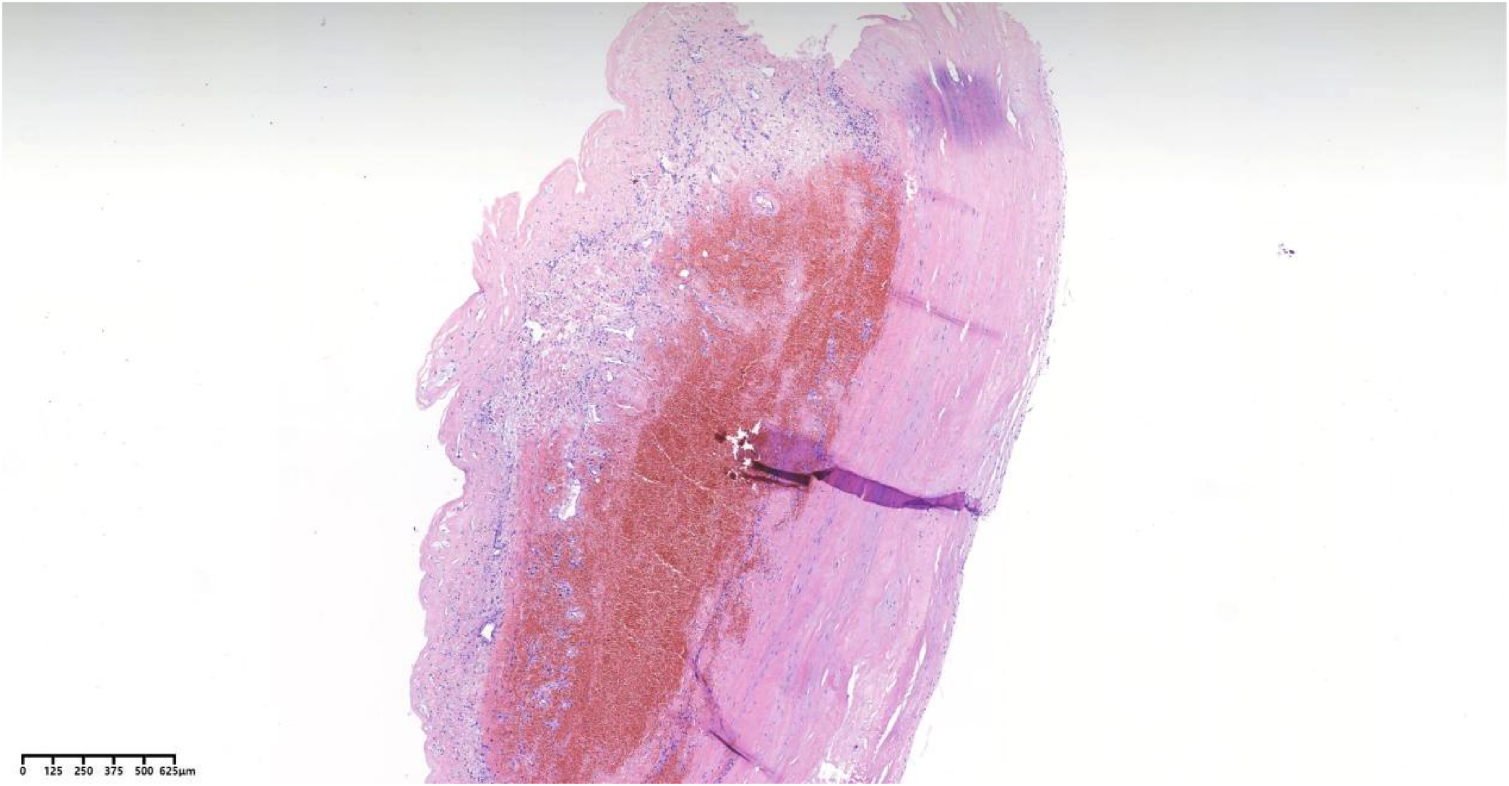
Pathological analysis employing hematoxylin-eosin staining reveals vascular wall fibrous hyperplasia, hyaline degeneration, and thrombus formation.

Following meticulous deliberation, surgical intervention is deemed necessary considering the risk of aneurysm rupture. The procedure was conducted through a median sternotomy, meticulously achieving hemostasis in a stepwise manner, followed by a gradual opening of the pericardium, revealing an accumulation of approximately 100 mL of aged, dark red hemopericardium, concomitant with partial thrombus formation (Figure 3). Remarkably, a substantial aneurysm was discerned at the aortic root and atrioventricular groove, measuring approximately 12 cm × 10 cm (Figure 4). Establishment of cardiopulmonary bypass ensued, achieved through cannulation of the ascending aorta and femoral vein. Following cardiac arrest, the aneurysm was meticulously incised for exploration, unveiling a 1.5 cm × 2 cm communication between the right coronary sinus wall and the aneurysm, with the right coronary artery ostium positioned 5 mm proximal to the opening (Figure 5). Intraoperatively, in light of the operative perils entailed in aneurysm extirpation and aortic root reconstruction, and judiciously considering the prospective postoperative outcomes, it was decided to employ a 2.5 cm × 2.5 cm pericardial patch to wrap around the openings of the aneurysm at two sites, forming a conduit between them, facilitating the isolation of the remaining portion of the aneurysm while maintaining coronary blood flow (Figure 6). Subsequent to aortotomy, no notable hemorrhage emanated from the patch. Intraoperative transesophageal echocardiography confirmed the cessation of the aortic-to-aneurysm shunt. Successful weaning from cardiopulmonary bypass ensued, and thoracic closure ensued in accordance with established protocols. The duration of cardiopulmonary bypass was 67 min. Postoperative histopathological examination delineated fibrous tissue hyperplasia, hyalinization, and thrombus deposition, consistent with alterations characteristic of the “aneurysmal wall” (Figure 7).

**Figure 8:**
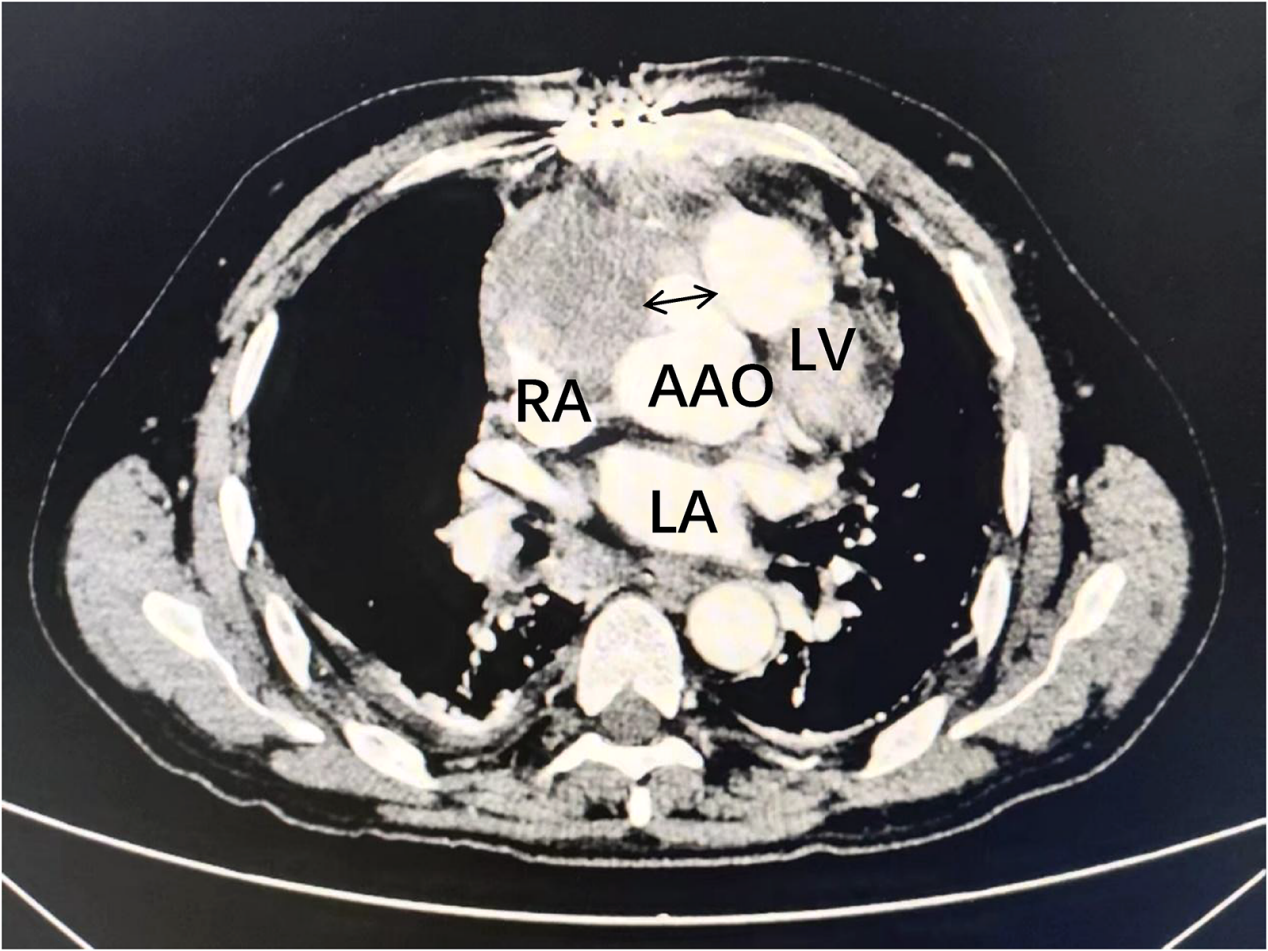
Computed tomography angiography (CTA) suggests localized nodular protrusions in the right coronary sinus, measuring approximately 1.4 × 1.2 cm (black arrow). LA, left atrium; LV, left ventricle; RA, right atrium; AAO, ascending aorta.

**Figure 9:**
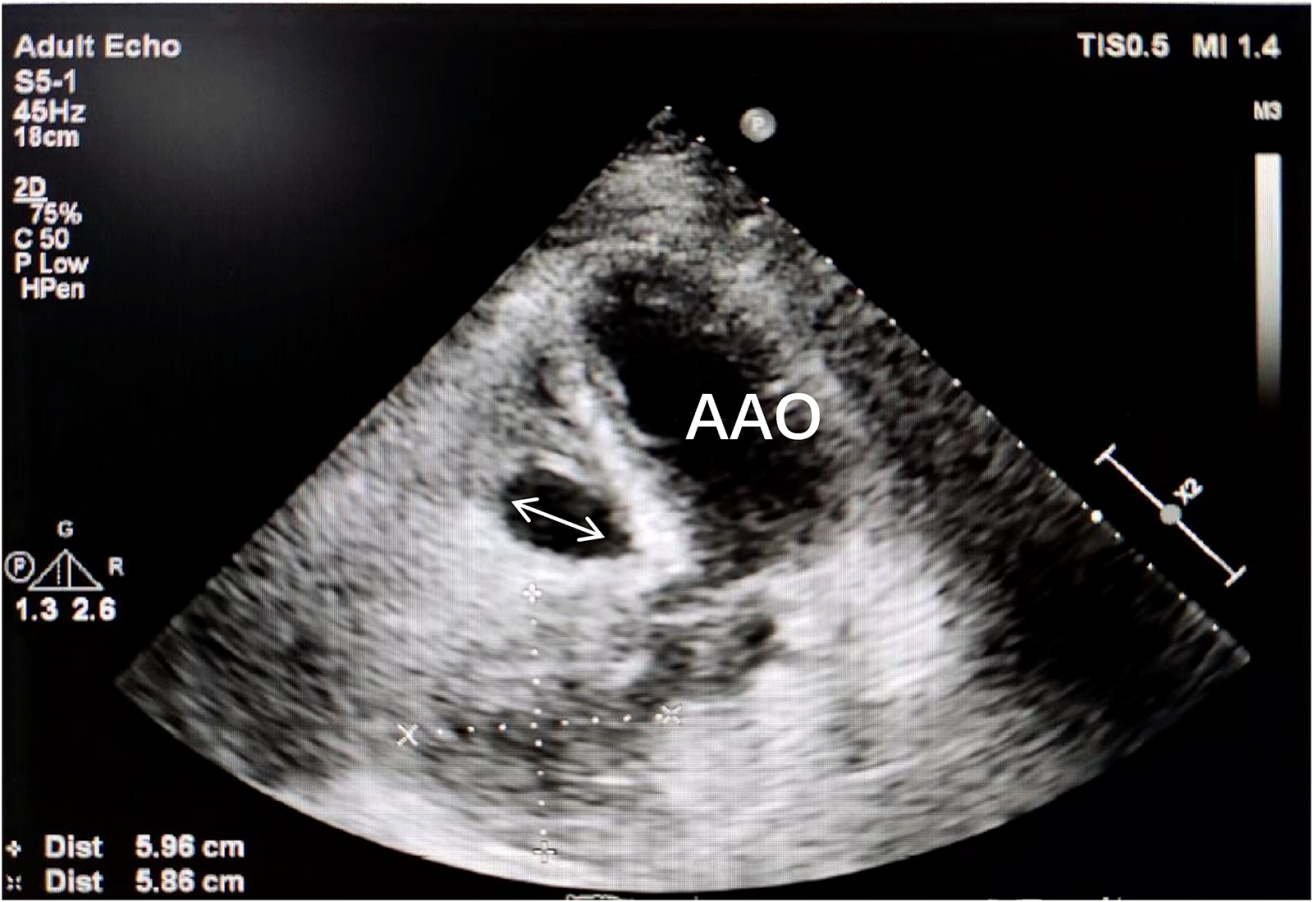
Transthoracic echocardiography (TTE) demonstrated continuous echogenicity along the wall of the aortic sinus to the right coronary artery, indicative of thrombosis within the residual lumen (white arrow) of the right coronary artery aneurysm.

One month postoperative, the patient attended a follow-up appointment at our institution. Computed tomography angiography (CTA) revealed localized nodular protrusions in the right coronary sinus, measuring approximately 1.4 cm × 1.2 cm (Figure 8). Transthoracic echocardiography (TTE) demonstrated continuous echogenicity along the wall of the aortic sinus to the right coronary artery, indicative of thrombosis within the residual lumen of the right coronary artery aneurysm (Figure 9).

## Discussion

Coronary artery aneurysms (CAAs) can often remain asymptomatic, yet their symptomatic manifestation frequently involves acute coronary syndrome or respiratory distress (6). Conversely, GCAAs demonstrate diverse and occasionally ambiguous clinical presentations, occasionally resembling mediastinal masses or cardiac neoplasms (7).

In this case, the patient's clinical course was brief, and the CT scan indicated a low-density fluid area surrounding the contrast-enhanced region. Pathological analysis further revealed fibrous hyperplasia, hyaline degeneration of the vessel wall, and thrombus formation. Consequently, we postulate that the patient developed a giant coronary artery pseudoaneurysm as a result of the traumatic injury (8).

In cases of coronary artery pseudoaneurysms, patient presentations can vary widely, ranging from asymptomatic to myocardial infarction, ventricular arrhythmias, or respiratory symptoms (9). In this report, the rapid formation of a coronary artery pseudoaneurysm is considered to have led to cardiac compression, resulting in transient cardiac insufficiency accompanied by loss of consciousness.

The management of CAAs should be individualized based on the patient's unique clinical presentation. When underlying primary diseases are present, therapeutic strategies should primarily address these underlying conditions. Specific treatment modalities for CAAs encompass both interventional and surgical interventions, necessitating a thorough evaluation of factors including aneurysm dimensions, anatomical location, and etiological factors (10). The treatment approach for coronary artery pseudoaneurysms is similar to that for coronary artery aneurysms, but it requires additional consideration of the heightened risk of pseudoaneurysm rupture (11, 12). In the context of this case report, the proximity of the coronary artery ostium to the aneurysmal opening raises concerns regarding potential obstruction of the coronary artery ostium with interventional embolization. Consequently, surgical exploration and repair of the opening are deemed appropriate and efficacious.

## Conclusion

Giant coronary artery pseudoaneurysms are relatively rare and have diverse etiologies. Therefore, treatment for giant coronary artery pseudoaneurysms needs to be individualized based on the patient's condition, addressing both the underlying primary disease and the aneurysm itself. Currently, there is a lack of sufficient evidence to support specific treatment strategies for post-traumatic giant coronary artery pseudoaneurysms, highlighting the need for further research.

## Data Availability

The raw data supporting the conclusions of this article will be made available by the authors, without undue reservation.

## References

[1] Pham V Hemptinne Q Grinda JM Duboc D Varenne O Picard F. Giant coronary aneurysms, from diagnosis to treatment: a literature review. Arch Cardiovasc Dis. (2020) 113(1):59–69. 10.1016/j.acvd.2019.10.008 31866173

[2] Eshtehardi P Cook S Moarof I Triller HJ Windecker S. Giant coronary artery aneurysm: imaging findings before and after treatment with a polytetrafluoroethylene-covered stent. Circ Cardiovasc Interv. (2008) 1(1):85–6. 10.1161/CIRCINTERVENTIONS.107.763656 20031659

[3] Ko K Kroeze V Heijmen RH Verkroost M Smith T. Surgical treatment of a giant right coronary aneurysm. Multimed Man Cardiothorac Surg. (2024) 2024. 10.1510/mmcts.2023.099 38376439

[4] Morita H Ozawa H Yamazaki S Yamauchi Y Tsuji M Katsumata T A case of giant coronary artery aneurysm with fistulous connection to the pulmonary artery: a case report and review of the literature. Intern Med. (2012) 51(11):1361–6. 10.2169/internalmedicine.51.7134 22687842

[5] Dhakam S Ahmed H Jafferani A. Percutaneous coronary intervention of left main pseudoaneurysm with customized covered stents. Catheter Cardiovasc Interv. (2011) 77(7):1033–5. 10.1002/ccd.22903 21413127

[6] Arboine L Palacios JM. Left main coronary artery aneurysm. N Engl J Med. (2018) 378(23):e32. 10.1056/NEJMicm1708877 29874527

[7] Dadkhah Tirani H Aghajanzadeh M Pourbahador R Hassanzadeh R Ebrahimi H. Giant right coronary artery aneurysm mimicking a mediastinal cyst with compression effects: a case report. Res Cardiovasc Med. (2016) 5(3):e32086. 10.5812/cardiovascmed.32086 27800453 PMC5075429

[8] Yoshioka D Izutani H Ryugo M Kawachi K Sawa Y. Asymptomatic giant traumatic right coronary artery pseudoaneurysm caused by sternal fracture. Ann Thorac Surg. (2011) 92(2):e33–5. 10.1016/j.athoracsur.2011.03.046 21801898

[9] Aqel RA Zoghbi GJ Iskandrian A. Spontaneous coronary artery dissection, aneurysms, and pseudoaneurysms: a review. Echocardiography. (2004) 21(2):175–82. 10.1111/j.0742-2822.2004.03050.x 14961799

[10] Zeb M McKenzie DB Scott PA Talwar S. Treatment of coronary aneurysms with covered stents: a review with illustrated case. J Invasive Cardiol. (2012) 24(9):465–9. 22954568

[11] Kar S Webel RR. Diagnosis and treatment of spontaneous coronary artery pseudoaneurysm: rare anomaly with potentially significant clinical implications. Catheter Cardiovasc Interv. (2017) 90(4):589–97. 10.1002/ccd.26997 28258964

[12] Wang YC Hsu RB Huang CH. Spontaneous giant right coronary artery pseudoaneurysm. J Thorac Cardiovasc Surg. (2014) 148(1):349–50. 10.1016/j.jtcvs.2013.12.023 24485964

